# Fibropapillomatosis and the Chelonid Alphaherpesvirus 5 in Green Turtles from West Africa

**DOI:** 10.1007/s10393-021-01526-y

**Published:** 2021-07-09

**Authors:** Jessica Monteiro, Margarida Duarte, Kidé Amadou, Castro Barbosa, Nahi El Bar, Fernando M. Madeira, Aissa Regalla, Ana Duarte, Luís Tavares, Ana Rita Patrício

**Affiliations:** 1 Centro de Investigação Interdisciplinar em Sanidade Animal (CIISA), Faculdade de Medicina Veterinária, Universidade de Lisboa, Avenida da Universidade Técnica, 1300-477 Lisboa, Portugal; 2 Instituto Nacional de Investigação Agrária e Veterinária (INIAV), 1500-310 Lisboa, Portugal; 3 Observatoire, Parc National du Banc d’Arguin, Chami, Mauritania; 4 Instituto da Biodiversidade e das Áreas Protegidas, Av. Dom Settimio Arturro Ferrazzetta, CP 70 Bissau, Guinea-Bissau; 5 cE3c Centre for Ecology, Evolution and Environmental Changes, Faculty of Sciences, University of Lisbon, Campo Grande 016, I749‐016 Lisboa, Lisboa Portugal; 6 MARE – Marine and Environmental Sciences Centre, ISPA – Instituto Universitário, Rua Jardim do Tabaco 34, 1149-041 Lisbon, Portugal; 7 Centre for Ecology and Conservation, University of Exeter, Penryn, TR10 9EZ UK

**Keywords:** Emerging diseases, Fibropapillomatosis, *Chelonia mydas*, Chelonid alphaherpesvirus 5, ChAHV5, Guinea-Bissau, Mauritania

## Abstract

Fibropapillomatosis (FP) is a tumorigenic panzootic disease of sea turtles, most common in green turtles (*Chelonia myd*as). FP is linked to the chelonid alphaherpesvirus 5 (ChAHV5) and to degraded habitats and, though benign, large tumours can hinder vital functions, causing death. We analyse 108 green turtles, captured in 2018 and 2019, at key foraging grounds in Guinea-Bissau and Mauritania, West Africa, for the presence of FP, and use real-time PCR to detect ChAHV5 DNA, in 76 individuals. The prevalence of FP was moderate; 33% in Guinea-Bissau (*n* = 36) and 28% in Mauritania (*n* = 72), and most turtles were mildly affected, possibly due to low human impact at study locations. Juveniles had higher FP prevalence (35%, *n* = 82) compared to subadults (5%, *n* = 21), probably because individuals acquire resistance over time. ChAHV5 DNA was detected in 83% (*n* = 24) of the tumour biopsies, consistent with its role as aetiological agent of FP and in 26% (*n* = 27) of the ‘normal’ skin (not showing lesions) from FP turtles. Notably, 45% of the asymptomatic turtles were positive for ChAHV5, supporting multifactorial disease expression. We report the first baselines of FP and ChAHV5 prevalence for West Africa green turtles, essential to assess evolution of disease and future impacts of anthropogenic activities.

## Introduction

In the past two decades, the human population has grown exponentially surpassing seven billion individuals in the year 2019 (United Nations 2019), leading to increased interactions between humans and wildlife, and to higher anthropogenic pressure on natural ecosystems, threatening the long-term survival of several species (Deem 2015). Climate change, habitat degradation and infectious diseases are major threats to biodiversity and ecosystems, which can have synergistic effects on one another (Hoegh-Guldberg et al. 2010). Some infectious diseases with known major impacts on wildlife populations are chytridiomycosis in amphibians (Scheele et al. 2017), canine distemper in carnivores (Kennedy et al. 2000) and fibropapillomatosis in sea turtles (Work et al. 2004).

Sea turtles, being long-lived organisms that display fidelity to their feeding and breeding habitats, can be used as sentinel indicators of the health of marine ecosystems (Aguirre and Lutz 2004; Domiciano et al. 2017). Fibropapillomatosis (FP) is a tumorigenic disease that has been reported in all seven species of sea turtles; however, it is more frequent among green turtles (*Chelonia mydas*). It was first reported in 1938 in a captive green turtle (Smith and Coates 1938), and ever since, it has been documented in sea turtle aggregations worldwide (Jones et al. 2016).

Fibropapillomas may be found on the flippers, the soft skin around the carapace, the ocular and oral regions and in extreme cases, the internal organs (Jones et al. 2016). Tumours can cause difficulties in sight, feeding and swimming and cause internal pressure, which may culminate in organ dysfunction and/or physiologic imbalances (Page-Karjian 2019). The aetiology of the disease, although not fully understood, is linked to an alphaherpesvirus, the chelonid alphaherpesvirus 5 (ChAHV5, Domiciano 2019), as its DNA is found in most FP lesions (Alfaro-Núñez and Gilbert 2014; Herbst et al. 2004). ChAHV5 DNA has also been detected in grossly FP-free turtles, suggesting that the development of tumours is not due to virus infection alone, but other factors may be implicated, such as host–pathogen–environment interactions, host immunity, viral load and severity of virus variant (Herbst et al. 2008). Phylogenetic analysis estimates that modern variants of ChAHV5 appeared before the widespread FP phenomenon (Patrício et al. 2012), suggesting that recent outbreaks are linked to human-induced environmental changes. Some studies reported higher FP prevalence in human-altered environments (Van Houtan et al. 2010; Keller et al. 2014), further supporting this hypothesis.

The prevalence of the disease and its severity differs spatially and temporally (Patrício et al. 2016), with some reports of prevalence peaks surpassing 90%, leading to high mortality (dos Santos et al. 2010). There is an age-effect on disease prevalence; juveniles are more affected (Jones et al. 2016; Patrício et al. 2016). Regression of the tumours has been reported (Aguirre et al. 1999, Bennett et al. 1999, Limpus et al. 2005; Hirama and Ehrhart 2007; Chaloupka et al. 2009; Machado Guimarães et al. 2013), and cycles of epidemic outbreaks followed by regression may be common (Patrício et al. 2016).

Geographically, there are major gaps on the knowledge of FP prevalence, particularly in West Africa. Lesions compatible with fibropapillomas have been observed in stranded green turtles from Mauritania, Senegal, The Gambia, Guinea-Bissau (Girard 2015) and Cape Verde (Martins et al. 2020), and Catry et al. (2009) found no evidence of FP among nesting green turtles in Guinea-Bissau. However, to this date, there were no assessments of FP on foraging animals in the region. Even in the wider Eastern Atlantic, only one study to date characterized FP and the infection of ChAHV5 among diseased and asymptomatic foraging green turtles, from the Principe Island, Gulf of Guinea (Duarte et al. 2012). In West Africa, the two main feeding sites for green turtles are Guinea-Bissau and Mauritania. The Bijagós Archipelago, in Guinea-Bissau, hosts the third largest green turtle population in the Atlantic Ocean, sixth worldwide, with an average of 25,436 nests per year (2013–2016, Patrício et al. 2018), as well as important foraging grounds for immature green turtles. In Mauritania, the Banc D’Arguin National Park supports high levels of marine productivity and a key foraging area for the reproductive green turtles from the Bijagós and for immature green turtles (Cardona et al. 2009; Godley et al. 2010). Although their origin is unknown, most likely, a great proportion of these immature turtles originates from the large rookery in the Bijagós (Patrício et al. 2017).

Guinea-Bissau and Mauritania are key ‘hotspots’ for the green sea turtle in the Atlantic Ocean; however, until this date, no data were available concerning the prevalence of FP, or the prevalence of infection by the ChAHV5, in foraging turtles from either of these sites. To support local conservation efforts and contribute to fill-in identified geographic information gaps, we i) assessed the presence, severity and prevalence of FP, ii) investigated whether ChAHV5 infection was ubiquitous among symptomatic and asymptomatic individuals, and iii) explored the relationship between FP and body size (a proxy for age), on foraging green turtles from West Africa.

## Materials and Methods

### Study Site

**Figure 1:**
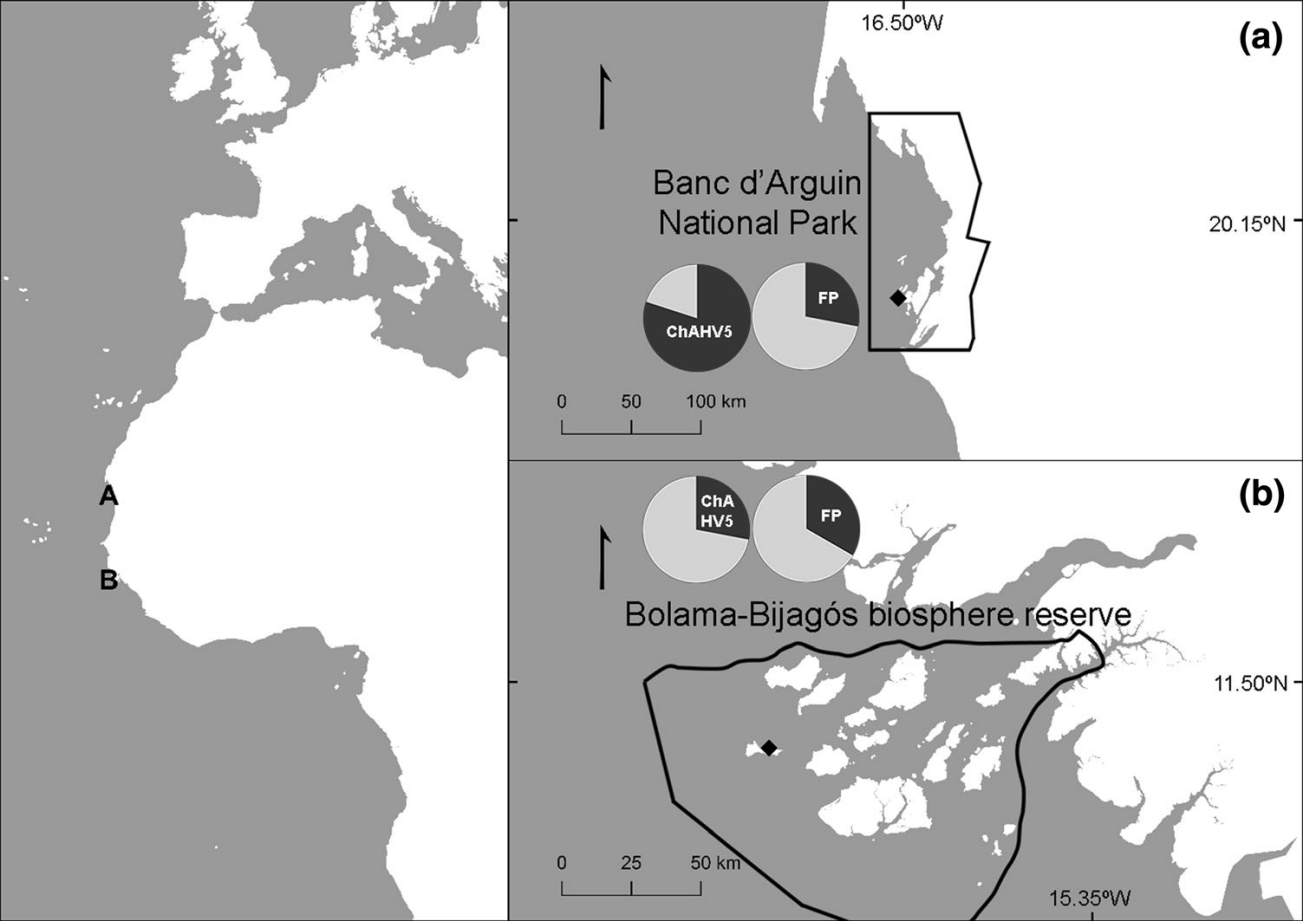
Location of green turtle foraging grounds, in the coast of West Africa, assessed in the present study. **a** Capture locations (black diamond) within the National Park of the Banc d'Arguin (PNBA), Mauritania (polygon shows park limits); **b** Unhocomo and Unhocomozinho (U&U, black diamond) and limits of the Bolama-Bijagós Biosphere Reserve (polygon). In each panel, the pie charts indicate the prevalence of fibropapillomatosis (FP) and prevalence of chelonid herpesvirus 5 (ChAHV5), linked to FP disease.

This study was conducted at two sites, the south of the National park of the Banc D’Arguin, in Mauritania (PNBA, N19.58°, W16.42°, Fig. 1), and the westernmost islands of the Bijagós Archipelago in Guinea-Bissau, Unhocomo and Unhocomozinho (U&U, N11.31°, W16.40°, Fig. 1). At the PNBA, the mean annual sea surface temperature is 20.3 °C (ranging from 17.8 °C to 22.7 °C). At U&U, sea surface temperature average is 27.3 °C (ranging from 25.1 °C to 29.5 °C).

### Capture Methods

At the PNBA, two fieldtrips were held, in 8 May 2018 and 4–8 March 2019, and effort was 1.4 net sets.day^−1^. Upon observing turtles surfacing to breathe, the purse seine method was used to capture them, using three joined entangling nets (420 m long, 4 m deep, 32 cm mesh size), with the assistance of two sailing boats operated by local Imraguen fishermen. Captured turtles were processed on-board the fishing vessels. Three separate missions were held at U&U on 18–22 March 2018, 21–25 October 2018 and 25–28 March 2019; effort was 1 net set.day^−1^. Turtles were caught using one entanglement net (800 m long, 4 m deep, 20 m mesh size), deployed from a pirogue, operated by local Bijagós fishers, enclosing foraging sites for periods of 1 h, under constant monitoring for entangled turtles. Captured turtles were brought on-board a logistic vessel (anchored next to the net) for processing.

### Sample Collection and Tumour Scoring

For each animal, an identification photograph was taken of the head and post-orbital scales, and the curved carapace length (CCL) was measured with a flexible measuring tape, to the nearest 0.1 cm. Turtles were classified as juvenile (< 65 cm CCL), subadult (65–83 cm CCL) or adult (≥ 83 cm CCL). The adult size-class was established using the minimum size of nesting females from the nearby green turtle rookery at Poilão Island, Bijagós. Remaining size-classes followed the criteria published by Patrício et al. (2016), as there is no information on the somatic growth at these aggregations. For the lower age classes, the gender was not determined, given the absence of obvious sexual dimorphism before the adult life-stage. All turtles were observed for the presence of external fibropapillomas, throughout the surface of the body and in the ocular and oral regions. For a subset of 76 turtles, biopsies were collected for genetic detection of the ChAHV5. (We did not have biopsies of the 32 turtles captured during the last field season at the PNBA.) For each of these turtles, a sample of apparently ‘normal’ skin tissue (i.e. tissue with no visible signs of FP) was collected from the right shoulder, using a sterile biopsy punch (6 mm diameter), and, if lesions with the appearance of fibropapillomas—protruding pedunculated/verrucous and/or large external growths, ranging in colour from pale to pink, to pink greyish and black–were present, samples were collected from the most severe-looking lesion, from the external surface of the tumour punching approximately 0.5 cm deep with a sterile biopsy punch (6 mm diameter). For three individuals from U& U presenting ocular lesions compatible with fibropapillomas, sampling was not performed to avoid injury of the cornea. For all the biopsies, the region to be sampled was previously disinfected with a diluted povidone-iodine solution and new gloves were used to avoid cross-contamination. All samples were stored in 90% ethanol, in 2-ml screw cap microcentrifuge tubes with unique labels.

Each individual tumour was classified depending on the approximate diameter: Class A – < 1 cm; Class B–1 to 4 cm; Class C–4 to 10 cm; Class D – > 10 cm. Turtles were assigned a tumour score, from 0 to 3, based on the number of tumours in each class, following the classification by Work and Balazs (1999). The tumour scoring system reflects the spectrum of severity of gross FP lesions in green turtles; a score of 0 is attributed to non-afflicted turtles; 1 means lightly affected; 2, moderately; and 3 heavily afflicted (Work and Balazs 1999).

### Quantitative Polymerase Chain Reaction (qPCR) and Validation of ChAHV5 Detection

Extraction of total DNA was carried out with the DNeasy Blood & Tissue kit (Qiagen, Hilden, Germany), following manufacturers’ protocol for animal tissues. DNA quantification was performed using the NanoDrop™ 2000 spectrophotometer (Thermo Fisher Scientific, Wilmington, USA).

Screening of the samples for ChAHV5 DNA presence was accomplished by a quantitative PCR (qPCR), targeting an 86-bp highly conserved genomic region within the DNA polymerase gene (UL30) (Quackenbush et al. 2001), using the AccuStart II PCR SuperMix (2x) (QUANTABio). Reactions were run in a CFX96TM Optical Reaction Module (Bio-Rad). The cycling conditions were as follows: initial denaturation at 95 °C for 10 min, 45 cycles of 90 °C for 15 s and 60 °C for 1 min. A negative blank control was included in all PCR runs. The threshold cycle (Ct) value was registered.

To estimate the number of viral copies in each sample, the qPCR protocol described by Quackenbush et al. (2001) was implemented and validated to construct a standard curve. First, a plasmid DNA containing a 486-base pair long fragment from the ChAHV5 DNA polymerase gene was generated by the conventional PCR system described by VanDevanter et al. (1996) and cloned into the pGEM-T Easy Vector (Promega), and the recombinant plasmid was sequenced to confirm the insert was the desired partial ChAHV5 gene sequence. Triplicates of tenfold serial dilutions of the recombinant DNA ranging from 10^–1^ to 10^–11^ were prepared, quantified using the NanoDrop™ 2000 spectrophotometer, and tested. The standard curve was generated using the CFX Manager Software (Bio-Rad, USA).

The sensitivity of the qPCR method, expressed as the limit of detection (LOD), was determined by using the serial dilutions of the recombinant DNA (10^–1^ to 10^–11^). The last dilution where all three replicates gave a positive and specific amplification was considered as the LOD.

The reproducibility (inter-assay variability) of the method was tested by repeating the qPCR of the dilution series in three independent runs. The repeatability (intra-assay variability) of the method was tested by preparing three independent dilution series from 10^–2^ until 10^–6^ and subjecting to independent runs qPCR. The amplifications used the same conditions with the CFX96™ Optical Reaction Module (Bio-Rad).

### Statistical Analysis

The CCL values considering all size classes (Shapiro–Wilk test; W = 0.915, *p* = 8.388 × 10^–5^), the Ct values of apparently normal skin (W = 0.940, *p* = 0.031) and the Ct values of tumours (W = 0.873, *p* = 0.011) did not follow a normal distribution, so we used the nonparametric Mann–Whitney U test to assess whether there were significant differences in CCL and the Ct values (‘normal’ skin and tumours) between the two sites, and the Kruskal–Wallis test to assess whether there were significant differences in Ct values (‘normal’ skin and tumours) between life-stages (juvenile, subadult and adult) and between tumour scores. The CCL values excluding the adult turtles followed a normal distribution (Shapiro–Wilk test; W = 0.97962, p-value = 0.113); thus, we used a parametric T-test to assess whether there were significant differences in the CCL of immature turtles between sites. We explored the relationship between FP presence and CCL at each study site, using generalized additive modelling (GAM) implemented with the package mcgv (Wood and Wood, 2015) in RStudio (RStudioTeam 2018). GAMs are semiparametric models that allow nonlinear relationships between the response variable (FP presence) and explanatory variables (turtle size, which is a proxy for age). An alpha level of 0.05 was used for all statistical tests.

## Results

### Fibropapillomatosis Prevalence and Tumour Score

**Figure 2:**
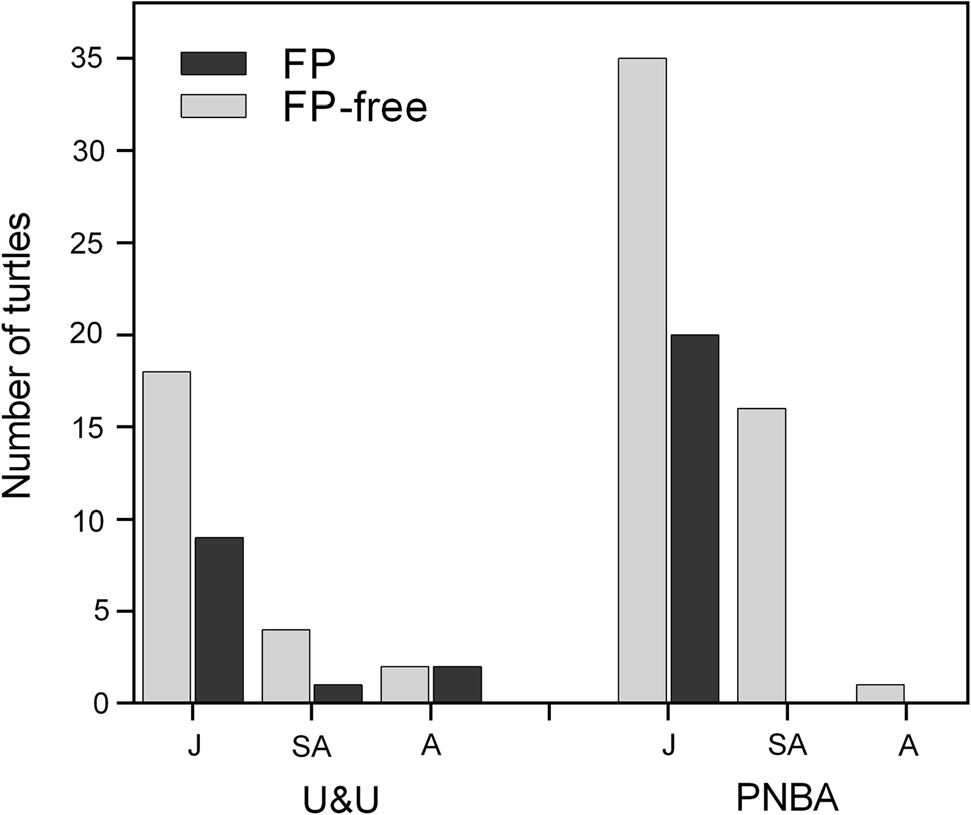
Number of green turtles captured at foraging sites in Unhocomo and Unhocomozinho, Guinea-Bissau (U&U), and in the National Park of the Banc d'Argin, Mauritania (PNBA), distributed by life-stage (J: juveniles, CCL < 65 cm; SA: subadults, 65 ≤ CCL < 83 cm; A: adults, CCL ≥ 83 cm). CCL: curved carapace length. Turtles with fibropapillomatosis (FP) are shown in dark grey, turtles free of FP lesions are shown in light grey.

**Figure 3:**
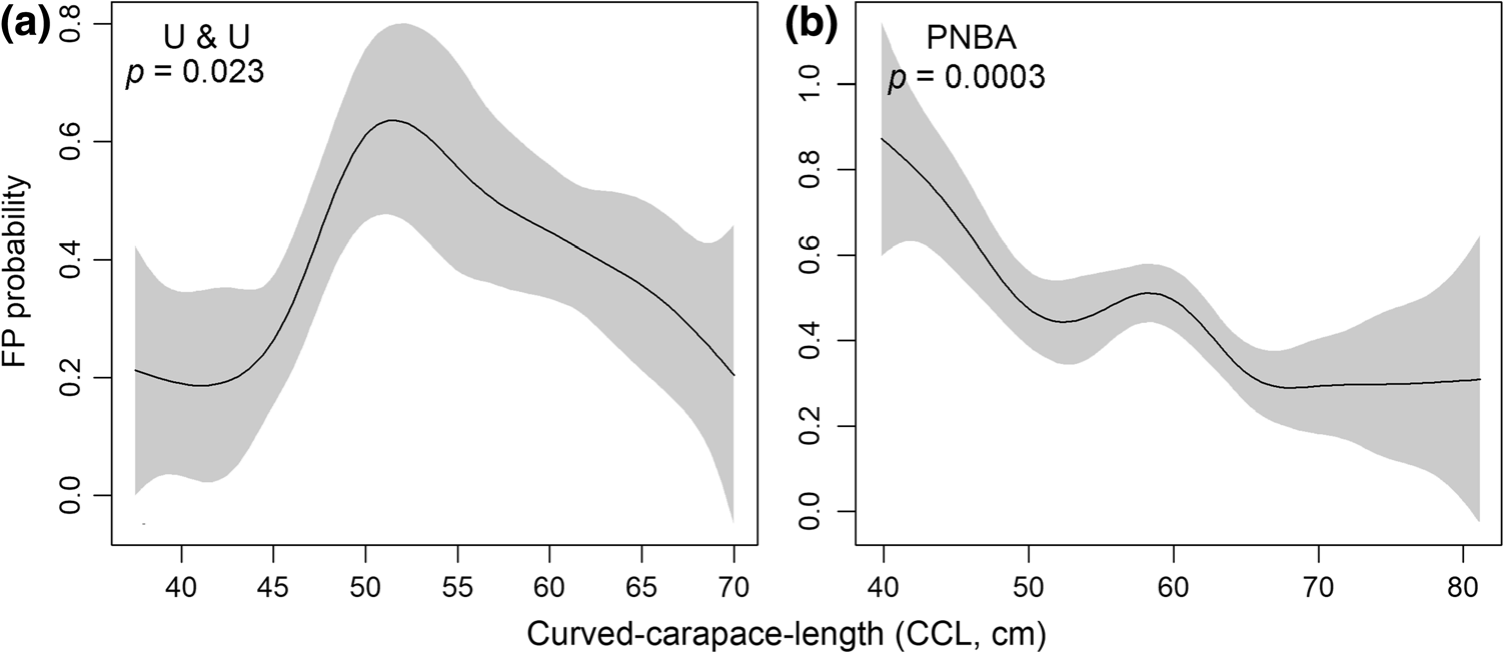
Graphical summary of generalized additive models fitting the relationship between curved carapace length (CCL, cm) and presence of fibropapillomatosis (FP) among immature green turtles foraging at two sites in West Africa: Unhocomo and Unhocomozinho, Guinea-Bissau (U&U), and in the National Park of the Banc d'Argin, Mauritania (PNBA). Response variable: probability of FP. Predictor variable: CCL.

In total, 108 green turtles were examined for the presence and severity of FP: 36 were captured in U&U, and 72 at the PNBA. At both sites, most turtles belonged to the juvenile size class (Fig. 2). At U&U, four adults were captured (two males and two females), ranging in CCL from 87.5 to 97 cm, while at the PNBA only one adult female was captured with 96 cm CCL. There was no significant difference in CCL between sites (W = 1101, *p* = 0.205) when considering all size-classes, and the mean CCL of individuals from U&U was 58.24 ± 15.3 cm (mean ± *SD*, range: 37.5 to 97 cm), slightly lower than the mean CCL at the PNBA, which was 59.52 ± 9.44 cm (ranging from 39.7 to 96 cm). Considering only immature-sized individuals, there was a significant difference between sites (t = -2.5103, df = 52.775, *p* = 0.015), with mean CCL at U&U being smaller (54.0 ± 9.8 cm) than at the PNBA (59.0 ± 8.4 cm). Of the 108 captured animals, 32 (30%) exhibited gross lesions compatible with FP (Table 1). The mean CCL of individuals presenting gross FP lesions was 55.9 ± 11.13 cm (ranging from 49.0 to 93.0 cm) in U&U, and 52.5 ± 7.1 cm (ranging from 39.7 to 61.6 cm) at the PNBA. The minimal adequate GAMs showed that the CCL was significantly correlated with FP risk at both sites (U&U: *p* = 0.023, PNBA: GAM, *p* = 0.0003), at U& U the probability of developing FP was lower for smaller individuals then increasing and peaking for turtles with CCL between 50–55 cm, decreasing thereafter ( Fig. 3a). At the PNBA, the smaller turtles had higher FP probability, but there was also a small peak for mid-sized juveniles, around 60 cm CCL (Fig. 3b). The adult size-class was excluded from our analysis due to the reduced sample size (n = 5), which could bias results. Overall, most FP turtles were only mildly afflicted, as only four turtles (12.5%) had a tumour score of 3, but even these seemed to present a good body condition (i.e. not emaciated), and the tumours did not seem to be affecting locomotion or any vital function (Table 2).

**Table 1:** Prevalence of Fibropapillomas (FP) and ChAHV5 DNA Among Green Turtles Foraging at the Coastal Waters of Unhocomo and Unhocomozinho Islands (U&U), in Guinea-Bissau, and the Banc d’Arguin National Park (PNBA), in Mauritania, and Prevalence of ChAHV5 at Each Location by Tissue Type.

Parameter	U & U	PNBA
FP presence	12/36 (33.3%)	20/72 (28.0%)
ChAHV5 presence	10/36 (28.0%)	32/40* (80.0%)
ChAHV5 in 'normal' skin of FP-free turtles	5/24 (20.8%)	17/25 (68.0%)
ChAHV5 in 'normal' skin of FP-afflicted turtles	1/12 (8.3%)	6/15 (40.0%)
ChAHV5 in FP tumours	5/9** (55.6%)	15/15 (100%)

*At the PNBA, biopsies of 40 out of the 72 turtles were analysed for viral presence (see methods)

**At U&U three turtles with tumours in the eyes were not samples to avoid injury (see methods)

**Table 2:** Number of Foraging Green Turtles Partitioned by Study Site and Life-Stage, Within Each Tumour Score (TS).

Site	TS	Life-stage		
		Juvenile	Subadult	Adult
U&U	0	18	4	2
	1	8	0	1
	2	0	0	0
	3	1	1	1
PNBA	0	35	16	1
	1	18	0	0
	2	1	0	0
	3	1	0	0

U&U: Unhocomo and Unhocomozinho islands, Bijagós, Guinea-Bissau. PNBA: Banc d’Arguin National Park, Mauritania. TS0 = FP absence, TS1 = mild FP, TS2 = moderate FP, TS3 = severe FP (Work and Balazs 1999). FP: fibropapillomatosis

### ChAHV5 Prevalence

**Figure 4:**
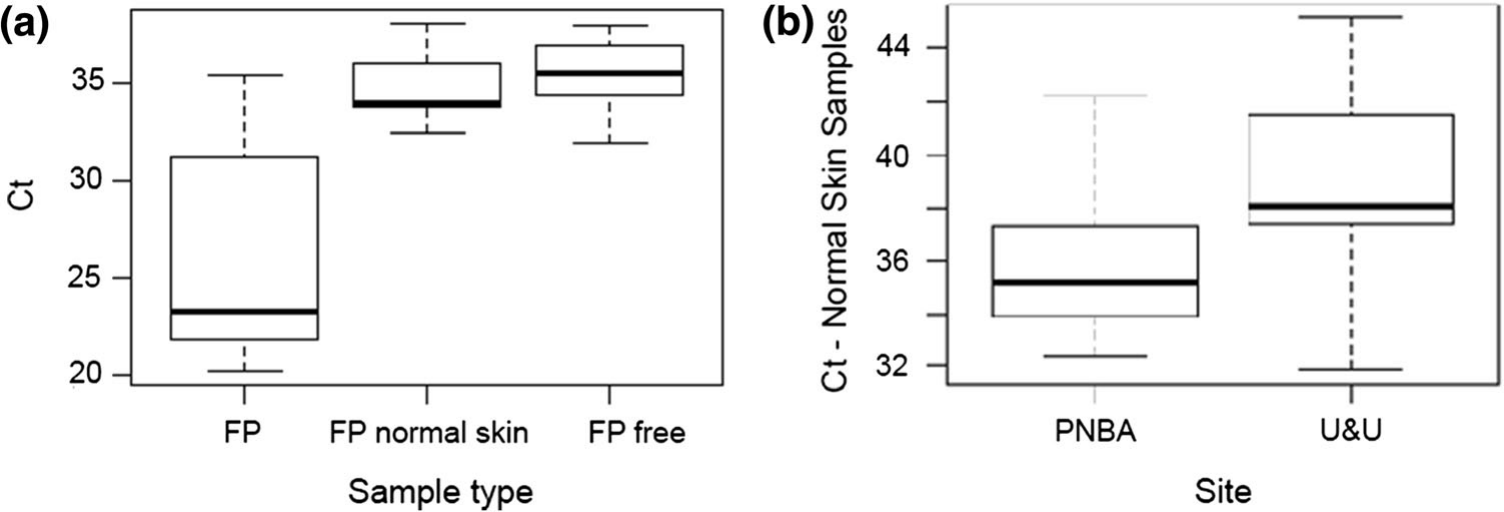
Ct values in different samples from foraging green turtles: **a** FP (fibropapilloma) samples (26.32 ± 4.26), ‘normal’ skin (free of FP lesions) from FP-afflicted animals (34.70 ± SD) and ‘normal’ skin from FP-free turtles (35.50 ± 1.87)—study sites pooled; **b** ‘normal’ skin samples from FP-free green turtles from the Banc d’Arguin National Park, Mauritania (PNBA, 34.01 ± 1.75) and from Unhocomo and Unhocomozinho islands, Guinea-Bissau (U&U, 36.21 ± 3.73). Boxes indicate median, upper and lower quartiles, whiskers indicate highest and lowest observation. FP: fibropapillomatosis.

In total, 76 individuals were analysed for the presence of ChAHV5 DNA; 36 from U&U (12 of these FP-afflicted and 24 FP-free), and 40 from the PNBA (15 FP afflicted, 25 FP-free). At the PNBA, one tumour sample was analysed from each of the 15 FP-afflicted turtles, while at U&U we analysed one tumour sample from each of nine FP-afflicted turtles, as three turtles were not sampled to avoid injury (see methods). Overall, a total of 83.3% (20 out of 24) of the tumour biopsies were positive for the ChAHV5 DNA, but there were differences between sites: all of the tumours from PNBA were positive to viral detection, compared to approximately half of the tumours at U&U (Table 1). The viral copy loads per mg within tumour tissues ranged from 4.25 × 10^2^ to 1.51 × 10^7^, with a mean of 3.99 × 10^6^ ± 4.52 × 10^6^ copies. The presence of ChAHV5 DNA was also detected in 25.9% (7 out of 27) of the apparently normal skin from FP-afflicted turtles and in 44.9% (22 out of 49) of the normal skin samples from asymptomatic turtles, but again this was highly variable between sites (Table 1). As expected, the number of viral load (ChAHV5 DNA copies) was higher in tumour samples compared to samples of apparently normal skin tissue (Fig. 4). There was no statistically significant difference in the Ct values of tumour samples between tumour scores (χ^2^ = 0.162, df = 1, *p* = 0.688), nor between life-stages (χ^2^ = 1.3561, df = 2, *p* = 0.5076). Similarly, we found no significant differences in the Ct values of ‘normal’ skin either between tumour score (χ^2^ = 1.414, df = 1, *p* = 0.234) or between life-stages (χ^2^ = 1.728, df = 2, *p* = 0.422). No statistical association was found between the Ct obtained for the tumour samples and the geographic locations (W = 62, *p* = 0.205); however, the viral loads found in the normal skin samples were significantly different between sites (W = 262, *p* = 0.005), with higher Ct values, corresponding to lower viral loads observed in samples from U&U (Fig. 4).

### Quantitative Polymerase Chain Reaction (qPCR) and Validation of ChAHV5 Detection

The DNA concentration yielded from both normal and tumour skin samples varied between 6.9 and 51.7 ng/µl. For turtles where both these samples were collected, the amount of DNA obtained from tumours was, in general, higher than from normal skin (mean ± SD = 38.11 ± 5.85 ng/mL and 18.34 ± 19.48 ng/mL, respectively). The mean DNA quantity obtained from normal skin was 17.44 ng/mL, while from tumours was 38.11 ng/mL. The standard curve generated had 100% amplification efficiency (slope = 3.322) and a high correlation coefficient (*r*^2^ = 0.995). The LOD of the qPCR method was five copies of viral DNA (1.8 × 10^−17^ g) deducted from the standard curve.

## Discussion

Aside from the present, there are no studies reporting FP prevalence among foraging green turtles in the region of West Africa. The prevalence of FP found in this study was higher than that reported at two green turtle foraging sites in Central Africa: Loango Bay, Congo, and Corisco Bay, Equatorial Guinea, with mean FP prevalence values of 10% (2008–2014) and 16.98% (1998–2006), respectively (Girard 2016), and at one feeding site in East Africa: the Barren Islands, Madagascar (East Africa) with FP rates of 9-13% (2010–2012, Leroux 2010, Campillo 2011, 2012). Our values were more similar to the FP prevalence reported for green turtles from Príncipe Island, São Tomé and Principe (Central Africa), where 32% of juveniles (n = 25) and 36% of subadults (n = 22) were found to have FP (2009, Loureiro and Matos 2009). Compared to other sites worldwide, where anthropogenic impact is presumably higher, FP prevalence at our study sites seems to be moderate, e.g. Indian River Lagoon (Florida, USA) with prevalence up to 70% (Ehrhart et al. 2016), Puerto Manglar Bay (Puerto Rico) with prevalence up to 75% (Patrício et al. 2016) and Moreton Bay (Australia) with prevalence up to 70% (Aguirre et al. 2000). The sites where the turtles were captured for this study have low human presence, limited to artisanal fisheries and few local inhabitants. The PNBA is a marine-protected area with very low population density, where only local Imraguen people are allowed to fish, and using only traditional sailing boats (with no engine). The U&U islands have also low population density, concentrated in three small villages. The main local activities are small-scale fisheries engaged by local villagers and some migrant fishers from nearby West African countries. It is curious, therefore, that these remote areas, with different habitats and very limited anthropogenic impact, still hold ~ 30% FP prevalence, posing the question of what other factors contribute to disease expression.

At U & U FP probability peaked among individuals with ~ 50–60 cm in CCL, while at the PNBA, turtles with FP lesions were smaller, but a small peak was also observed at ~ 60 cm CCL. At both sites, after ~ 60-cm CCL FP probability decreased, similar to what was found at Loango Bay, Congo (Girard 2016). This trend of larger juveniles and subadults having lower probability of expressing tumours is consistent to what was found in a long-term capture-mark-recapture (CMR) study at a green turtle foraging aggregation in Puerto Rico (Patrício et al. 2016), and in a long-term study looking at stranded animals in Florida, USA (Foley et al. 2005), although at least one study reported higher FP prevalence among larger subadults (Baptistotte, 2007). Generally, the mean CCL of afflicted turtles was in accordance with previous studies (Balazs 1991; dos Santos et al. 2010; Foley et al. 2005; Patrício et al. 2016). The lower risk of lesions among larger individuals supports the hypothesis that green turtles may acquire immunity with age (Patrício et al. 2016). However, despite a low risk of having tumours being described for adults (Foley et. al, 2005), two of the four adults captured from U&U presented tumours. We captured very few (PNBA: n = 1; U&U: n = 3) ‘recruit’ size individuals (i.e. turtles with CCL < 40 cm, as per Patrício et al. 2014), but we know they exist in these locations, as we have found several stranded animals and old carapaces within the ‘recruit’ size-class, at both sites. Potentially, smaller turtles managed to escape the entanglement nets due to the large mesh sizes. Thus, we cannot infer if green turtles arrive at the foraging sites free of FP, or acquire the disease upon recruitment. Future surveys should adopt a technique to target this size-class.

Most FP-afflicted turtles (84.3%) were only mildly affected by the disease, with 12.5% classified as heavily afflicted. The development of FP has been linked to other co-infections and/or to immunological fragility, which may explain why some animals develop more severe symptoms (Herbst and Klein 1995). The heavily afflicted individuals may potentially have other increased risk factors, such as poorer nutritional condition or concomitant infections/diseases leading to a more severe expression of the clinical disease (Aguirre and Balazs, 2000). All turtles observed appeared to be in good body condition, yet we did not assess blood parameters, which would give a more complete picture of overall health condition, potentially explaining the observed differences in FP expression (Domiciano et al. 2019). The continuation of the in-water CMR monitoring will give more insight to the pathogenesis of the disease, as this study represents a snapshot in time, and there may be temporal variations in FP expression/severity, for example, mediated by seasonality in seawater temperatures, which are more variable in Mauritania, as warmer seawater temperatures have been proposed to be linked with increases in tumour growth (Herbst et al. 1994, Herbst et al. 1995, Foley et al. 2004). Indeed, at this site, more green turtles are found stranded in the warmer months (June and July, PNBA unpublished data), but we have no details on FP presence or severity among these stranded animals.

The detection of the ChAHV5 DNA in most tumour samples is consistent with the role of this virus in the aetiology of FP (Lu et al. 2000; Quackenbush et al. 1998) and further demonstrates that tumours are the best matrix for viral detection. However, not all samples were positive, which could be due to inhibitor factors, the stage of the tumour, relative low sensitivity of the qPCR technique used (Alfaro-Núñez and Gilbert 2014), or quality of the extracted DNA. A third of the samples from apparently normal skin of FP-afflicted turtles were positive for ChAHV5 DNA. Latency, a known ability of herpesviruses is an important point to address in ChAHV5 investigations. Research has shown that ChAHV5 has co-evolved with its host for at least 8.9 millions of years (Patrício et al. 2012), developing the ability, after establishing latency, of minimal viral expression to avoid detection by the host immune system (Alfaro-Núñez et al. 2016). During the active viraemic phase, viral DNA may be detected throughout the body, while in chronic infections viral activity may be limited to tumours (Alfaro-Núñez et al. 2014). This may lead to an unequal viral distribution across tissue types or body regions within the same individual and explain why, in most cases, only the tumours had high viral loads. Notably, there was no correlation between the tumour score and the Ct value in tumour samples. The number of viral copies could be more likely associated with tumour development stage, than to the severity of FP. For example, some of the tumours sampled from animals mildly afflicted (TS = 1) could be under rapid growth, with proliferation of host cells infected by ChAHV5, thus increasing the number of viral copies (Yetsko et al. 2020). Because our study represents a snapshot in time, we do not know, however, which turtles were developing tumours, which ones were in more stable diseased conditions or even undergoing tumour regression.

This study also revealed a relevant proportion of asymptomatic individuals (PNBA: 68.0%, U&U: 20.8%) infected with ChAHV5, similar to what has been found in other studies (Duarte et al. 2012, Page-Kajian et al. 2012, Alfaro-Núñez et al. 2014). This phenomenon was higher in turtles from the PNBA, Mauritania. The Ct values of the apparently normal skin samples were significantly lower in the turtles from Mauritania, indicating higher viral loads, compared to Guinea-Bissau turtles. This difference could indicate a variation in resistance to infection between the two foraging aggregations. Alternatively, FP outbreaks and prevalence may be more heavily influenced by environmental factors and/or host immunity than they are by viral circulation in the populations (Herbst et al. 2008). The fact that FP-free turtles are carriers of the virus shows that they are asymptomatic reservoirs, which may be undergoing either early or latent infection, or alternatively, tumour regression. In the cases of latency or early infection, these turtles may develop clinical disease in the future (Alfaro-Núñez et al. 2014; Quackenbush et al. 2001). The high prevalence of ChAHV5 in asymptomatic animals suggests that FP is enzootic at our study sites (Page-Karjian et al. 2020). Even though the evidence of reservoirs does not correlate to the prevalence of FP, it is important evidence to help understand the pathogenesis of the disease and supports its panzootic status (Herbst and Klein 1995; Herbst 1994).

### Final Considerations

Infectious disease outbreaks in marine ecosystems have been increasing, reactivating previously latent diseases and introducing new, potentially fatal ones (Cunningham et al. 2017). The implications of future climate change on FP are uncertain, but enhanced water temperatures as a result of global warming, and unbalanced ecosystems, have the potential to greatly increase the prevalence and virulence of wildlife diseases (Aguirre and Lutz 2004; Burge et al. 2014). Guinea-Bissau, in West Africa, is home to one of the largest green turtle populations globally (Patrício et al. 2019), with strong connectivity to foraging grounds in Mauritania (Godley et al. 2010). Although both the Guinea-Bissau and the Mauritania study locations seem to have very limited anthropogenic stressors, they had relevant FP prevalence values. Thus, to better understand the FP dynamics of West Africa green turtles, long-term monitoring is needed, as FP prevalence, and possibly its virulence, may fluctuate through time. This would increase sample size and allow the recapture of turtles, key to assess disease progression or regression. Thus, we recommend identifying turtles with flipper tags, commonly used in marine turtle monitoring programs (NOAA, 2008). This study reports for the first time the presence of infection by the ChAHV5 in West Africa green turtles, and it establishes a baseline for the prevalence of FP and of ChAHV5 in two key areas for this species.
